# Anesthetic Management of a Patient With Symptomatic May-Thurner Syndrome: A Case Report

**DOI:** 10.7759/cureus.91302

**Published:** 2025-08-30

**Authors:** Matthew J McIntyre, Caroline Nikolaidis, Gisele J Wakim

**Affiliations:** 1 Department of Anesthesiology, Perioperative Medicine, and Pain Management, University of Miami Miller School of Medicine, Miami, USA; 2 Department of Anesthesiology, Perioperative Medicine, and Pain Management, Jackson Memorial Hospital, Miami, USA

**Keywords:** anticoagulation, deep vein thrombosis (dvt), may-thurner syndrome (mts), pulmonary embolism (pe), total intravenous anesthesia (tiva), vascular compression, venous stent, volatile anesthetics

## Abstract

May-Thurner syndrome (MTS) is characterized by the compression of the left common iliac vein by the overlying right common iliac artery, which can lead to venous insufficiency, obstruction, and an increased risk of iliofemoral deep vein thrombosis (DVT) and pulmonary embolism (PE). We report the perioperative anesthetic management of a 38-year-old female with symptomatic MTS who underwent a total laparoscopic hysterectomy, bilateral salpingectomy, and lysis of adhesions for abnormal uterine bleeding. The patient had a history of persistent left lower extremity symptoms despite prior left common iliac vein stenting and was on chronic anticoagulation therapy with rivaroxaban. Given her history of severe postoperative nausea and vomiting (PONV), total intravenous anesthesia (TIVA) with propofol and dexmedetomidine was utilized, along with standard induction agents and antiemetic prophylaxis. Invasive arterial monitoring was employed due to her elevated thromboembolic risk, and intermittent pneumatic compression devices were applied. The patient tolerated the procedure without complications and was restarted on rivaroxaban at discharge on postoperative day two. This case highlights key perioperative considerations in patients with MTS, including thromboembolic and bleeding risks, the timing of anticoagulation cessation and resumption, and the implications for anesthetic technique. Although no definitive evidence exists favoring one anesthetic technique over another in MTS, the use of TIVA may offer theoretical benefits that need to be researched further. In addition, the use of intraoperative measures to maintain normothermia and euvolemia was prioritized to mitigate bleeding risk. This case underscores the importance of individualized anesthetic planning and multidisciplinary collaboration when managing patients with symptomatic MTS undergoing surgery.

## Introduction

May-Thurner syndrome (MTS) is characterized by the compression of the left common iliac vein (LCIV) by the overlying right common iliac artery (RCIA) against the fifth lumbar vertebrae. The prevalence of MTS in the literature is variable and dependent on the population that is studied; however, one study found that 61.3% of patients with left-sided ileofemoral deep vein thrombosis (DVT) had MTS [1]. MTS is also known to be more prevalent in females [2]. Many patients with MTS are asymptomatic; however, compression of the LCIV can lead to venous insufficiency, obstruction, and stenosis [3]. The constant pulsatile stress from the RCIA leads to endothelial damage and impaired venous drainage [3]. MTS can also lead to iliofemoral DVT and pulmonary embolism (PE) [3]. In addition, diagnosed MTS accounts for about 2-5% of all cases of DVT [3-6].

Patients with MTS who are asymptomatic can develop symptoms following a precipitating event, such as pregnancy, prolonged immobilization, surgery, and during the post-partum period [6]. Symptomatic patients can undergo endovascular stenting of the LCIV. Some patients experience symptoms even after stenting and are often placed on chronic anticoagulation therapy [7]. There is currently no consensus on whether anticoagulation or antiplatelet therapy following endovascular stenting for a non-thrombotic iliac vein lesion is necessary [8]. Anticoagulation regimens are often tailored to each patient's individual situation [8]. The main perioperative risks surrounding an MTS patient undergoing surgery include perioperative DVT or PE and excessive bleeding risk, depending on the patient’s coagulation status and the type of operation being performed [3]. There is currently no recommended anesthetic management for patients with MTS in the literature.

This case was previously presented as a poster at the Florida Society of Anesthesiologists 2025 Annual Meeting on June 7, 2025.

## Case presentation

**Figure 1:**
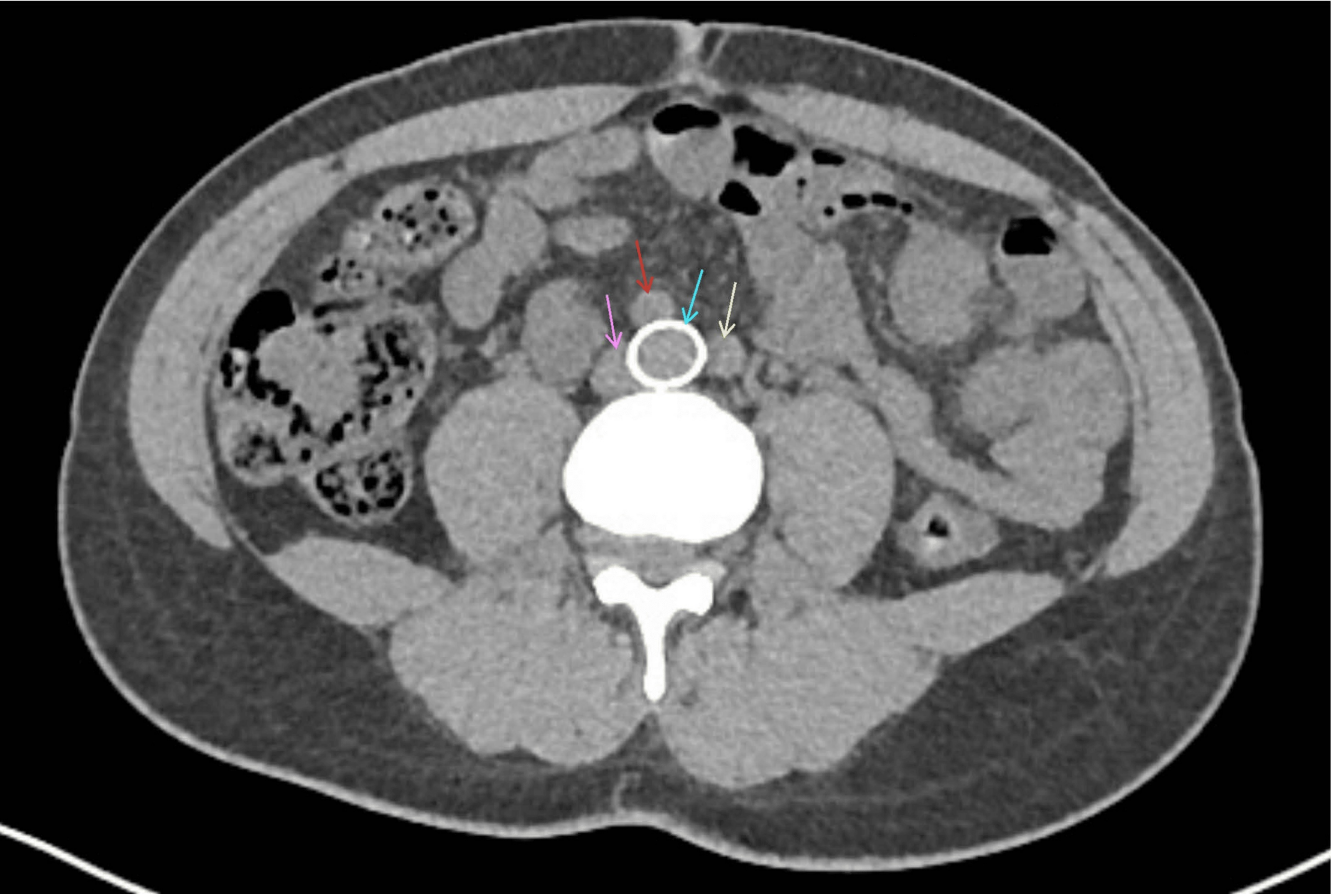
Axial CT view of the RCIA, LCIA, RCIV, and stented LCIV. CT: computed tomography; RCIA: right common iliac artery; LCIA: left common iliac artery; RCIV: right common iliac vein; LCIV: left common iliac vein The pink arrow shows the RCIV. The red arrow shows the RCIA. The blue arrow shows the stented LCIV. The yellow arrow shows the LCIA.

**Figure 2:**
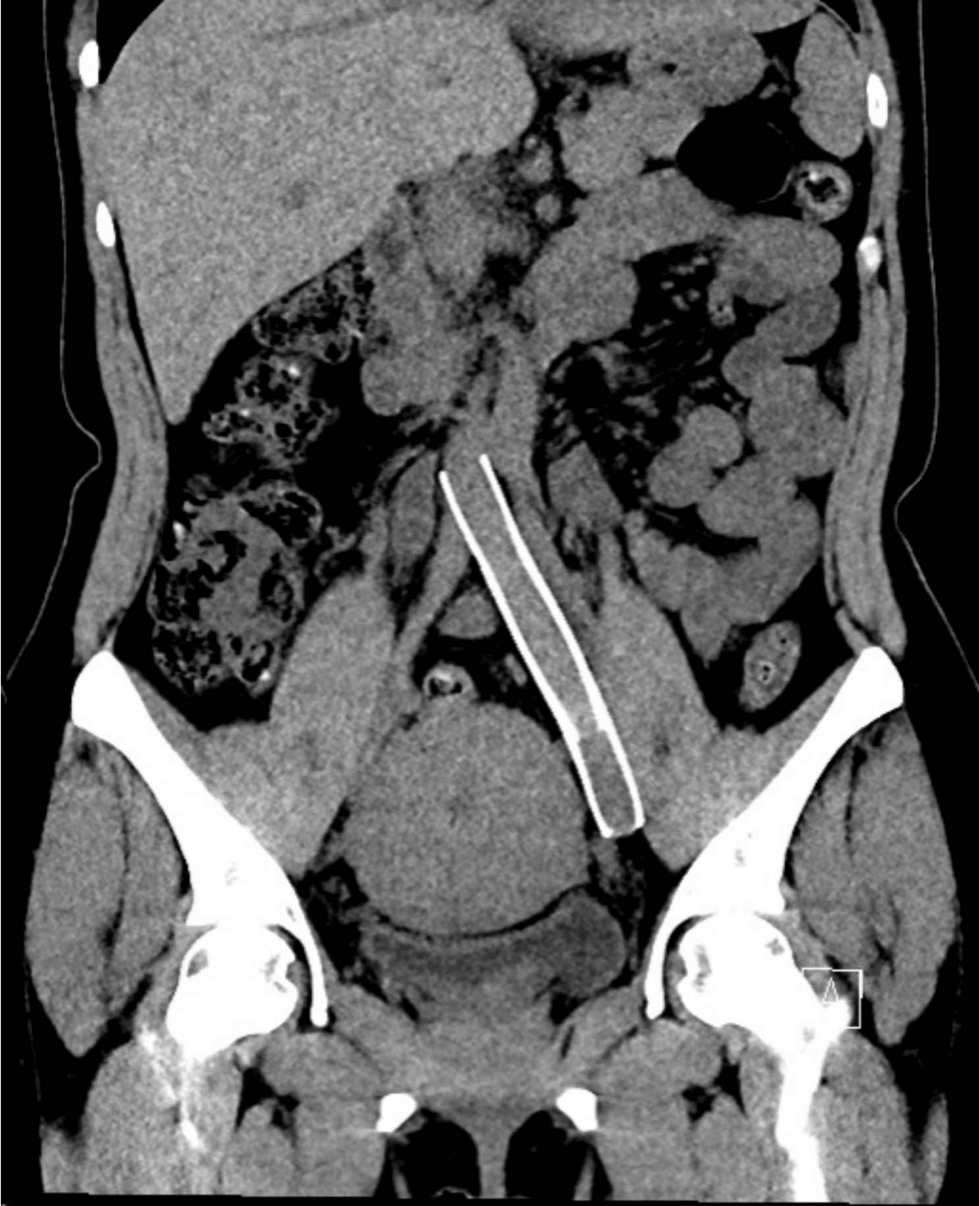
Coronal CT view of the stented LCIV. CT: computed tomography; LCIV: left common iliac vein

**Figure 3:**
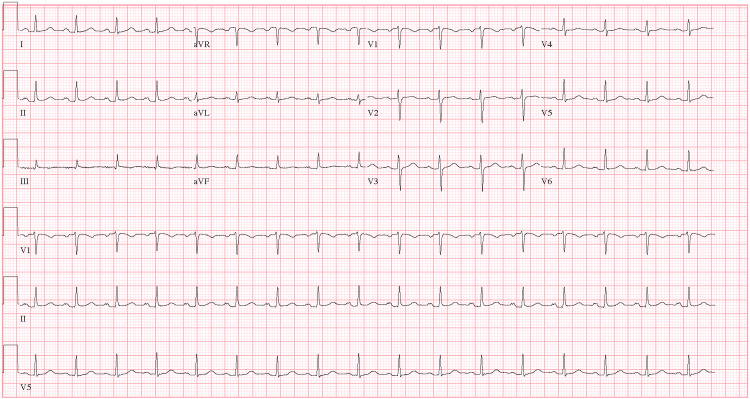
Preoperative electrocardiogram showing sinus tachycardia with otherwise normal findings. aVR: augmented vector right; aVL: augmented vector left; aVF: augmented vector foot

We present the case of a 38-year-old female, ASA II, 170 cm, 74 kg, BMI 25.6, with a history of MTS who presented for a total laparoscopic hysterectomy, bilateral salpingectomy, and lysis of adhesions for abnormal uterine bleeding secondary to fibroids. Medical history was significant for symptomatic MTS diagnosed at age 20 with persistent left lower extremity pain, swelling, and hyperpigmentation, without a history of DVT or PE. Other medical history included asthma, anemia, and severe PONV. She received an LCIV stent seven years prior to the current presentation and a stent revision four years prior to the current presentation for persistent symptoms. She has had no previous perioperative complications. Computed tomography scans following stent revision in Figures 1-2 show a patent LCIV stent with no acute thrombosis. Her medications included albuterol as needed and rivaroxaban 20 mg nightly for anticoagulation, which was stopped seven days before surgery without low-molecular-weight heparin bridging at the recommendation of her vascular surgery team. The patient endorsed good exercise tolerance with metabolic equivalents greater than four and no functional limitations. Preoperative labs are shown in Table 1. Preoperative EKG, shown in Figure 3, revealed sinus tachycardia.

**Table 1:** Preoperative labs WBC: white blood cell count; HgB: hemoglobin; Hct: hematocrit; Plt: platelets; Na^+^: sodium; K^+^: potassium; CO2: bicarbonate; Cl^-^: chloride; BUN: blood urea nitrogen; Cr: creatinine; Glu: glucose; PT: prothrombin time; aPTT: activated partial thromboplastin time; INR: international normalized ratio; FEU: fibrinogen equivalent units

Lab	Preoperative value	Reference range	Unit
WBC	5.0	4.0–11.0	x10³/µL
HgB	11.5	12.0–16.0	g/dL
Hct	36.5	36–44	%
Plt	275	150–400	x10³/µL
Na⁺	137	135–145	mEq/L
K⁺	4.6	3.5–5.0	mEq/L
CO₂	28	22–29	mEq/L
Cl⁻	104	95–105	mEq/L
BUN	14	7–20	mg/dL
Cr	0.90	0.6–1.3	mg/dL
Glu	93	<140	mg/dL
PT	12.9	11–13.5	seconds
aPTT	27	25–35	seconds
INR	0.98	0.8–1.2	ratio
D-Dimer	0.34	< 0.5	μg/mL FEU

We proceeded with general anesthesia with a size 7.0 endotracheal tube. Given the patient’s history of severe PONV, we utilized total intravenous anesthesia (TIVA) using propofol (140 mcg/kg/min) and dexmedetomidine (0.7 mcg/kg/hour), and standard induction with intravenous fentanyl (1.25 mcg/kg), propofol (2.5 mg/kg), and rocuronium (0.6 mg/kg). The patient also received intravenous dexamethasone (8 mg) and ondansetron (8 mg) for PONV. Standard monitors were used, including a temperature probe, pulse oximetry, end-tidal CO_2_, non-invasive blood pressure, and a five-lead electrocardiogram. A bispectral index (BIS Quatro) was used to monitor anesthetic depth. The patient's Caprini VTE score was four points, indicating moderate risk [9]. Due to the patient’s increased risk of a thromboembolic event, a right radial arterial line was placed to monitor hemodynamic instability. The patient was induced without complications. The patient was ventilated on assist-control/volume-control mode with a tidal volume of 500 mL (6.75 mL/kg), respiratory rate of 15, fraction of inspired oxygen of 40%, and a positive end-expiratory pressure of five cm. Lower extremity intermittent pneumatic compression devices were utilized. A Bair Hugger (3M) was used to maintain body temperature above 35 degrees Celsius. She received a balanced crystalloid solution for fluid maintenance. Activated clotting time at the beginning and end of the procedure was elevated at 148 and 150 seconds, respectively (Table 2). The patient tolerated the anesthesia and surgery well, with no intraoperative or perioperative complications. The total duration of the surgery was just over four hours. She received intravenous hydromorphone for post-operative pain control on post-op day one. She was transitioned to oral hydromorphone on post-op day two, followed by oral acetaminophen upon discharge. She was discharged on post-op day two and was restarted on rivaroxaban 20 mg upon discharge. Follow-up visits at two weeks and six months showed adequate recovery with no complications.

**Table 2:** Preoperative and end-of-surgery activated clotting times ACT: activated clotting time The initial value was at the first incision of the surgery. The final value occurred near the end of the procedure. The reference range is for patients not on any anticoagulation medications.

Lab	Initial value	Final value	Reference range	Unit
ACT	148	150	70-120	seconds

## Discussion

The main perioperative risks for a patient with MTS undergoing surgery include the development of a DVT/PE and excessive blood loss [3]. The surgical team, including the anesthesiologist, must achieve a balance of anticoagulation that prevents both DVT/PE and excessive bleeding. Patients with MTS in the lithotomy position are at even greater risk of DVT due to increased compression of the LCIV during hip flexion [10]. A significant sign of intraoperative PE is a decrease in the end-tidal CO_2_ on the ventilator. For patients who are taking direct oral anticoagulants (DOACs), the American College of Cardiology (ACC) and the American Heart Association (AHA) 2024 guidelines state that patients undergoing a procedure with a low/moderate bleeding risk should withhold the DOAC one day prior to surgery and should resume taking the DOAC on post-op day one [11]. Minimal, low/moderate, and high bleeding risks are categorized as having a 30-day risk of major bleeding of 0%, <2%, and >2% respectively. In the case of our patient undergoing a laparoscopic total abdominal hysterectomy, bilateral salpingectomy, and lysis of adhesions, this procedure is categorized as having low/moderate bleeding risk. Therefore, our patient could have continued to take rivaroxaban until the day before her scheduled surgery to optimize her risk of bleeding and DVT/PE perioperatively. Even after stopping rivaroxaban seven days prior to the procedure, the patient had elevated ACTs, as shown in Table 2. Clinical judgement is sometimes preferred for patients with complex histories [11].

In determining the anesthetic plan for the patient, the choice of general, regional, and neuraxial anesthesia must be determined, depending on the type of procedure being performed. According to the AHA/ACC, there is no clear clinical difference between regional and general anesthesia in terms of risk of an adverse cardiovascular event [11]. However, there is controversy regarding the difference between regional anesthesia and general anesthesia in hip fracture patients. Some meta-analyses have shown a decrease in the risk of DVTs in patients receiving regional anesthesia for hip fracture surgery [12]. This finding is thought to be explained by a reduction in sympathetic tone to the lower limbs or through altered blood viscosity and coagulability [13,14]. Other meta-analyses have shown no such differences between general and regional techniques [15]. Regardless, neuraxial anesthesia is contraindicated for those on anticoagulation therapy due to the risk of epidural/spinal hematoma formation. As a result, many patients with symptomatic MTS who are on anticoagulation therapy cannot receive neuraxial anesthesia.

In patients with MTS who will be receiving general anesthesia, the question remains whether there is any clinical difference between volatile anesthetics and TIVA. Similar to the risk of regional versus general anesthesia, the AHA/ACC guidelines state that there is no evidence that volatile anesthetics or TIVA confer an advantage in perioperative outcomes [11]. Considering that these guidelines do not encompass the circumstances of every patient, different anesthetic techniques may confer differences in perioperative risk among patients with symptomatic MTS. For example, sevoflurane has been shown to increase plasma P-selectin and ICAM-1, markers of endothelial inflammation, while TIVA with propofol has not shown any effect on these markers [16]. Furthermore, a study showed that patients undergoing TIVA in knee arthroplasty had a lower incidence of DVT compared to patients receiving volatile anesthesia [17]. For our patient, TIVA with propofol was definitively indicated due to her history of severe PONV; however, it is worth considering the possible advantage that TIVA could provide to patients with symptomatic MTS. Patients in this population already have endothelial damage and increased venous stasis, and it is possible that further insult to the blood vessel endothelium could be clinically relevant. More research is needed to determine if there are any significant differences among different anesthetic techniques in patients with MTS.

Other methods of optimizing perioperative risk for patients with MTS include temperature and fluid management. Normothermia and euvolemia were maintained throughout the surgery. Hypothermia is known to increase intraoperative blood loss and increase transfusion requirements [18]. The Restrictive versus Liberal Fluid Therapy in Major Abdominal Surgery (RELIEF) trial in 2018 showed that for patients at increased risk for complications during major abdominal surgery, a restrictive perioperative fluid regimen led to increased rates of acute kidney injury [19]. However, individualized fluid management is likely necessary to maintain euvolemia in patients.

## Conclusions

This case discusses the anesthetic management of a mechanically hypercoagulable patient secondary to MTS. Although neuraxial anesthesia may confer an advantage over general anesthesia depending on the procedure, many patients with MTS who are on anticoagulation regimens cannot receive neuraxial anesthesia due to the risk of spinal/epidural hematoma. In addition, a careful balance of anticoagulation is required to prevent hypercoagulability and excessive bleeding risk. The AHA/ACC guidelines offer specific timelines for when to discontinue and restart anticoagulation based on the type of anticoagulation being taken and the procedure being performed. Invasive monitoring with an arterial line is also necessary due to the risk of DVT and PE. Although TIVA was definitively indicated for the patient’s PONV, it is worth considering TIVA over volatile anesthetics in patients with MTS due to the potential increase in thrombus formation secondary to endothelial damage with sevoflurane use. Moreover, adequate temperature and fluid management to achieve normothermia and euvolemia are important to optimize patients’ perioperative risk.
